# Conversational AI and Vaccine Communication: Systematic Review of the Evidence

**DOI:** 10.2196/42758

**Published:** 2023-10-03

**Authors:** Aly Passanante, Ed Pertwee, Leesa Lin, Kristi Yoonsup Lee, Joseph T Wu, Heidi J Larson

**Affiliations:** 1 Department of Infectious Disease Epidemiology London School of Hygiene & Tropical Medicine London United Kingdom; 2 Laboratory of Data Discovery for Health Hong Kong Science Park Hong Kong China (Hong Kong); 3 WHO Collaborating Centre for Infectious Disease Epidemiology and Control School of Public Health, LKS Faculty of Medicine The University of Hong Kong Hong Kong China (Hong Kong); 4 Institute for Health Metrics and Evaluation University of Washington Seattle, WA United States

**Keywords:** chatbots, artificial intelligence, conversational AI, vaccine communication, vaccine hesitancy, conversational agent, COVID-19, vaccine information, health information

## Abstract

**Background:**

Since the mid-2010s, use of conversational artificial intelligence (AI; chatbots) in health care has expanded significantly, especially in the context of increased burdens on health systems and restrictions on in-person consultations with health care providers during the COVID-19 pandemic. One emerging use for conversational AI is to capture evolving questions and communicate information about vaccines and vaccination.

**Objective:**

The objective of this systematic review was to examine documented uses and evidence on the effectiveness of conversational AI for vaccine communication.

**Methods:**

This systematic review was conducted following the PRISMA (Preferred Reporting Items for Systematic Reviews and Meta-Analyses) guidelines. PubMed, Web of Science, PsycINFO, MEDLINE, Scopus, CINAHL Complete, Cochrane Library, Embase, Epistemonikos, Global Health, Global Index Medicus, Academic Search Complete, and the University of London library database were searched for papers on the use of conversational AI for vaccine communication. The inclusion criteria were studies that included (1) documented instances of conversational AI being used for the purpose of vaccine communication and (2) evaluation data on the impact and effectiveness of the intervention.

**Results:**

After duplicates were removed, the review identified 496 unique records, which were then screened by title and abstract, of which 38 were identified for full-text review. Seven fit the inclusion criteria and were assessed and summarized in the findings of this review. Overall, vaccine chatbots deployed to date have been relatively simple in their design and have mainly been used to provide factual information to users in response to their questions about vaccines. Additionally, chatbots have been used for vaccination scheduling, appointment reminders, debunking misinformation, and, in some cases, for vaccine counseling and persuasion. Available evidence suggests that chatbots can have a positive effect on vaccine attitudes; however, studies were typically exploratory in nature, and some lacked a control group or had very small sample sizes.

**Conclusions:**

The review found evidence of potential benefits from conversational AI for vaccine communication. Factors that may contribute to the effectiveness of vaccine chatbots include their ability to provide credible and personalized information in real time, the familiarity and accessibility of the chatbot platform, and the extent to which interactions with the chatbot feel “natural” to users. However, evaluations have focused on the short-term, direct effects of chatbots on their users. The potential longer-term and societal impacts of conversational AI have yet to be analyzed. In addition, existing studies do not adequately address how ethics apply in the field of conversational AI around vaccines. In a context where further digitalization of vaccine communication can be anticipated, additional high-quality research will be required across all these areas.

## Introduction

Since the mid-2010s, the use of conversational artificial intelligence (AI; chatbots) in health care has increased significantly, especially in the context of increased burdens on health systems and restrictions on in-person consultations with health care providers during the COVID-19 pandemic [1,2]. In response to these stresses on health systems, there has been a growing interest in how conversational AI and digital communication tools more generally can improve health-related knowledge, attitudes, and behaviors. Chatbots were already being used in a health context prior to the COVID-19 pandemic, primarily to assist with treatment and monitoring, patient education, health system support, behavior change, and diagnosis [3,4]. Uses of chatbots during the COVID-19 pandemic included (but were not limited to) triaging users based on their COVID-19 symptoms and risk factors, gathering data on disease symptoms and prevalence, disseminating information to the public, screening recovered patients for activities such as blood plasma donation, and aiding coordination and communication between health care workers and health organizations [1].

The association between chatbots and health communication dates back to the mid-1960s, when Joseph Weizenbaum developed the first chatbot, named ELIZA, which was used to simulate a consultation with a Rogerian psychotherapist [3,5]. Early chatbots like ELIZA were rules-based, meaning they used a series of preprogrammed rules to match user input to predefined outputs. More recent chatbots, such as Apple’s Siri or Amazon’s Alexa, use natural language processing (NLP) to parse user input and generate human-like responses. Relying on machine learning, these chatbots do not require predefined answers for all possible user inputs, and they are capable of “learning” from user input rather than being limited to the knowledge base they were programmed with [3]. The most sophisticated of these, such as ChatGPT and Google Bard, are based on large language models that are capable of responding to complex user queries across multiple knowledge domains, but these have yet to be widely adopted or evaluated within the health field. In addition to the broad distinction between rules-based and natural language bots, chatbots differ along a number of other dimensions, including the knowledge domain in which they operate (eg, health care, retail, and banking), the type of service they provide (eg, access to information, assisting with a task, and offering a service), the type of interface they use (eg, voice or text), the delivery channel (eg, website, smartphone app, social media channel, and SMS text message), and the extent to which they require human supervision [6].

One emerging use of conversational AI within the health field is to communicate information about vaccines and vaccination with the aim of building vaccine confidence [2]. In theory, a well-designed chatbot can disseminate accurate vaccine information in real time, assist users in finding available vaccination appointments, book appointments, issue appointment reminders, and address user concerns and questions about vaccines. The ability to provide timely and accurate information to the public at scale is particularly important in the context of what has come to be called an “infodemic,” characterized by the World Health Organization as an excess of “information including false or misleading information in digital and physical environments during a disease outbreak” [7]. Information ecosystem disorder is one of many threats to vaccine confidence and uptake and to public health more generally, resulting in a need for practical solutions that assist people in a context where information is abundant but not necessarily reliable. Proponents argue that chatbots are a potentially beneficial tool for this purpose, assuming they can provide real-time information from reliable and trustworthy sources on commonly used communication platforms. However, some previous research has raised concerns about the quality of health information provided by conversational AI [8-10].

Given the relatively recent application of chatbots in the context of vaccine communication, the evidence base around their potential uses and effectiveness in this field is still quite limited. In order to better understand the current state of knowledge in this area and identify ways forward, this systematic review aimed to (1) understand the current evidence base around the use of chatbots for vaccine communication and (2) identify key gaps in the evidence in order to suggest directions for future research. Our review contributes to the emerging literature on conversational AI in the health field [1,4,8-11]. Previous related work includes a scoping review [3] and 2 systematic reviews [10,11] of conversational AI within the health field as a whole; a number of domain- or disease-specific reviews (eg, chatbots focused on noncommunicable diseases, COVID-19, sexual health, and smoking cessation) [1,9,12,13]; and some technology-specific studies (eg, health information provided by voice assistants such as Siri and Alexa or via smartphone apps) [8,9,12].

In the following sections, we discuss the methodology for this review; key findings on vaccine chatbot design, use, and effectiveness; gaps and limitations in the available evidence; and recommendations for future research.

## Methods

### Search Strategy and Database Search

This methodology aims to identify and document recent vaccine-related chatbots and their impact on vaccine attitudes and behaviors. A keyword search strategy was used and applied across 13 databases (PubMed, Web of Science, PsycINFO, MEDLINE, Scopus, CINAHL Complete, Cochrane Library, Embase, Epistemonikos, Global Health, Global Index Medicus, Academic Search Complete, and a University of London library search). The search was applied across 13 databases to cast a wide net and ensure we did not miss any relevant literature. Three of these databases (CINAHL Complete, Cochrane Library, and Global Index Medicus) did not produce any relevant results, and others produced duplicates of literature already found on other databases, which increased confidence that we had hit a saturation point and found all of the relevant literature. The search was (vaccin* OR (immuniz* OR immunis*)) AND (chatbot OR “chat bot” OR “chat-bot” OR “conversational AI” OR “conversational artificial intelligence” OR “conversational agent” OR “conversational interface”). Relevant papers were identified and exported into an Excel (Microsoft Corp) spreadsheet.

### Screening and Selection of Papers

Two researchers (AP and EP) independently screened papers included in the Excel spreadsheet by title and abstract, and then by full text, according to the inclusion and exclusion criteria shown in Textbox 1. Full-text screening resulted in an agreement rate of 89%. Any remaining disagreements between the coders were resolved by discussion and mutual agreement.

**Inclusion and exclusion criteria.**

**Inclusion criteria**

- Focused on vaccine-related chatbots
- Attempted to evaluate effectiveness of chatbot in changing attitudes, behavior, or both
- Search included peer-reviewed papers, gray literature, and preprints

**Exclusion criteria**

- Not about vaccine-related chatbots (ie, addressed a different health-related issue or used a different web-based intervention)
- Did not attempt to evaluate a change in attitudes, behavior, or both (eg, only addressed feasibility of chatbot)

In contrast to some previous work [9], we preemptively decided to include studies that used a “Wizard of Oz” protocol, in which participants interact with what they believe to be an autonomous AI system but is actually an interface being controlled by a concealed human operator (the “wizard”). “Wizard of Oz” experiments are often used in the early phases of system design and testing to address design and usability issues before time and resources are invested in software development [10,13]. While the simulated conversational agents used in the “Wizard of Oz” experiments are not themselves autonomous AI systems, we nonetheless deemed them relevant since they contain data on how users perceive and interact with vaccine communications delivered by (what they perceive to be) autonomous AI systems.

We also chose to include preprints given that this is a rapidly evolving field and there may have been valuable evaluation insights that were not yet published. Similarly, we chose to include gray literature to allow for evidence produced outside of academia (eg, technology companies), as recent data suggest that current AI developments are primarily taking place in industry settings rather than academia [14]. However, we did not find any preprints or gray literature that met our other inclusion criteria at the time of writing, so only peer-reviewed papers are included.

### Data Extraction and Analysis

We recorded the following data for the various studies identified: authors, publication year, title, citation, abstract, location of study, vaccines studied, timeframe, aim, hypotheses, research design, and key findings. Given the heterogeneity of conversational agents, evaluation methods, and outcomes being measured, we opted to conduct a narrative synthesis rather than a meta-analysis.

## Results

Our literature search identified 971 records across 13 databases published before August 2022. We excluded 482 duplicates, and the remaining 496 were screened by title and abstract using the criteria listed above. We then screened out 426 records by title, leaving 70 to be reviewed by abstract. During the abstract screening, an additional 32 papers were excluded, leaving 38 for full-text review. From these, 31 were excluded for the following reasons: they did not discuss vaccine-related chatbots or evaluate the chatbot’s impact on attitudes, behaviors, or both.

**Figure 1:**
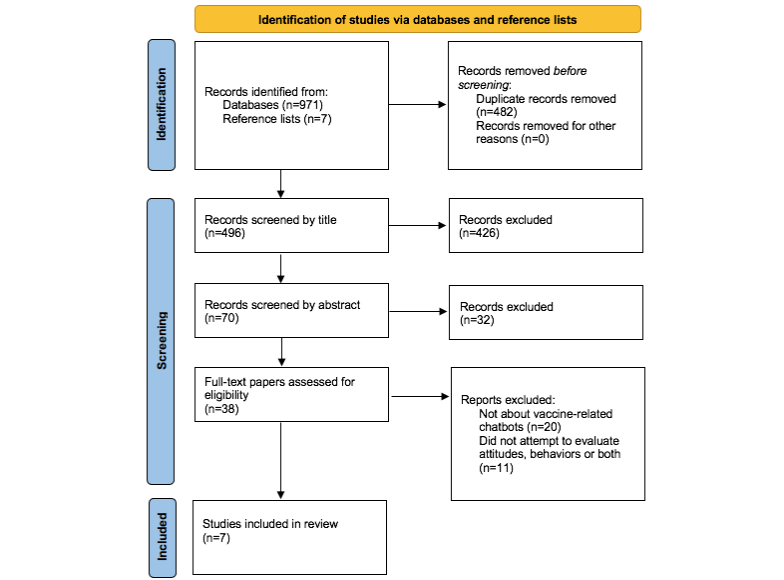
PRISMA (Preferred Reporting Items for Systematic Reviews and Meta-Analyses) paper search process flowchart (adapted from Page et al [15], which is published under Creative Commons Attribution 4.0 International License [23]).

At the time of our search, other vaccine-related chatbots were in development and missed our inclusion criteria either because they were still in the design phase or because they were evaluating feasibility or message content rather than impact on attitudes or behaviors. Given that this is a new and rapidly emerging research area, we expect that the relevant literature will increase quickly. However, at the time of this search, 7 papers fit the inclusion criteria and were assessed and summarized in the findings of this review. Seven additional papers from the reference lists of the 7 screened-in papers were also assessed as they appeared potentially relevant, but none of them met the inclusion criteria. Thus, 7 papers are included in this review. All 7 are peer-reviewed papers, and none are gray literature or preprints. This is purely because none of the gray literature or preprints identified in the search contained any evaluation data (Figure 1) [15].

Of the included publications, there were 3 studies in the United States [16-18], one each in France [19], South Korea [20], and Japan [21], and one study location was not explicitly stated but was inferred to be in the United Kingdom based on the institutional affiliations of the authors and mentions of the UK National Health Service within the text [22]. Three of the studies investigated COVID-19 vaccines [19,21,22], 3 evaluated human papillomavirus (HPV) vaccines [16-18], and one examined childhood immunizations (as recommended by the Republic of South Korea) [20]. However, there were only 6 unique chatbots, as 2 papers discussed the same chatbot at different points in its development cycle [16,17].

## Discussion

### Overview

The use of conversational AI in health care generally and for vaccine communication specifically is still an emerging field, and the state of the literature reflects this. In this section, we discuss the (1) design and uses of vaccine chatbots to-date, (2) evidence on their effectiveness, (3) user experience, and (4) key limitations and knowledge gaps. Due to the small number of studies identified by our literature search, we draw on the wider literature on health chatbots where appropriate to contextualize our findings.

### Chatbot Design and Use

Vaccine chatbots deployed to date have been relatively simple in terms of their design (Table 1). Out of the 6 unique chatbots identified by this review, 2 were NLP-based [20,22], a third was a hybrid with some NLP functionality integrated within a predominantly rules-based system [21], one was purely rules-based [19], and the remaining 2 were simulated agents (ie, “Wizard of Oz” experiments) [16-18]. Of the 3 chatbots that had some NLP capability, only one had the capability to generate natural language responses [20], while the other 2 were able to process natural language inputs but not generate natural language responses [21,22]. The dominance of relatively simple, rules-based bots is consistent with research on health chatbots more generally [1,4,10].

**Table 1:** Chatbot characteristics.

	Proprietary name	Country	Vaccines	Use cases	Chatbot type	Development platform	Deployment platform	Evaluation methodology	Outcome variables	Theoretical underpinning
Altay et al [19]	None	France	COVID-19	Information provision	Rules-based	Not specified	Custom-built web page	Randomized control trial (n=701)	COVID-19 vaccine attitudes; COVID-19 vaccine intent	None stated
Hong et al [20]	None	South Korea	Childhood vaccines as per national immunization schedule	Information provision; vaccine scheduling; appointment reminders; misinformation debunking; financial incentives	Natural language	Google Dialog-flow	Kakao Plus Friend	Quasi-experiment (n=65)	Vaccination information; Vaccination motivation; Self-efficacy; Vaccination behavioral intention	Information-motivation behavioral skills model
Chalaguine and Hunter [22]	None	United Kingdom (inferred)	COVID-19	Vaccine counseling or persuasion; information provision	Natural language	Javascript and Python	Flask web server	Experiment (n=300)	COVID-19 vaccine intent	None stated
Kobayashi et al [21]	Corowa-kun	Japan	COVID-19	Information provision	Hybrid rules-based or natural language	Not specified	LINE	Cross-sectional survey (n=10,192)	COVID-19 vaccine intent	None stated
Amith et al [16,17]	None	United States	HPV^a^	Vaccine counseling or persuasion; information provision	Simulated conversational agent (“Wizard of Oz”)	Apple SDK^b^	iPad app	Pre- and postuse surveys (2019: n=18; 2020: n=24)	Vaccine hesitancy; Perceived chatbot usability	Health Belief Model
Tsai et al [18]	None	United States	HPV	Information provision	Simulated conversational agent (“Wizard of Oz”)	tawk.to	Website	Experiment (n=142)	Satisfaction with chatbot; Perceived chatbot utility; HPV vaccine intent	Agency effect

^a^HPV: human papillomavirus.

^b^SDK: Software Development Kit.

Only 5 studies (4 unique chatbots) specified the platforms and programming languages used to develop their chatbots, which in those cases were Apple’s Software Development Kit [16,17], Google Dialogflow [20], tawk.to [18], and Python [22]. In terms of delivery platforms, 2 chatbots were provided via instant messaging services [20,21], a further 3 were hosted on custom-built web pages [18,19,22], while the sixth was delivered through an iPad app [16,17]. In most cases, the knowledge base for the chatbots was constructed from governmental websites and scientific literature, typically with review and verification of the answers by medical experts. Chatbot development was not generally informed by systematic analysis of local information environments prior to deployment, for example, by using social media and web search data to identify information-seeking behaviors or prevalent misinformation narratives among target populations. Only 3 chatbots (50%) had a theoretical underpinning to their approach [16-18,20], such as the Health Belief Model or Information-Motivation-Behavioral Skills Model (Table 1).

The main use for vaccine chatbots so far has been information dissemination, again consistent with research on health chatbots more generally [1]. All chatbots in the studies included in this review provided basic factual information to their users, such as data on vaccine safety and effectiveness and common side effects. Other uses included vaccination scheduling, appointment reminders, and infodemic management [20]. Some chatbots were also used for vaccine counseling or persuasion; that is, the chatbots proactively sought to persuade users to vaccinate themselves or their children rather than simply providing factual information and leaving users to make their own choice. In one case, the chatbot was programmed with a strong normative stance in favor of COVID-19 vaccination for its (adult) users [22]. Using NLP, the chatbot automatically identified the user’s concerns about COVID-19 vaccination based on their input and then provided counterarguments to persuade the user to get vaccinated. Other forms of persuasion included a protocol to pursue a recommendation in favor of HPV vaccination for their child in the event of (parental) user resistance or disengagement [16], and in another case, a financial incentive for parents to get their children vaccinated in the form of a drinks coupon [20].

### Effectiveness of Vaccine Chatbots

Like chatbots in other health domains [10], vaccine chatbots have not always been subject to robust evaluation. In addition to the 7 publications included in this review, a further eleven records that were identified through our literature search were excluded as there was no documented attempt to evaluate the chatbots described. Of the 6 unique chatbots that did meet the criteria, one had been evaluated using a randomized control trial [19], 3 through other experimental or quasi-experimental methodologies [18,20,22], one through a cross-sectional survey [21], and one using a pre- and postuse survey [16,17]. However, in many cases, the sample sizes were very small, ranging from 18 to 10,192, with a median sample size of 142. In all cases, evaluation was limited to the short-term, direct effects of chatbot use on users’ self-reported vaccine attitudes and behaviors, typically over a time period of days or at most a few weeks.

Notwithstanding these limitations, all the studies that sought to measure the influence of chatbots on users’ vaccine attitudes and behavioral intent found evidence of positive effects. None identified any “backfire effects” (where some participants become more vaccine hesitant after the intervention), which have been reported in some previous studies of digital health interventions [24-26]. However, one study did find some potential evidence that the relative benefits of chatbot use compared to nonuse may decline over time [19]. The results of the studies included in this review are not strictly comparable due to the use of slightly different attitudinal and behavioral metrics between studies and different operationalizations of these metrics within evaluation questionnaires. To enhance the comparability of future studies, consideration should be given to using standardized survey instruments in vaccine chatbot evaluation, such as the Chatbot Usability Questionnaire [27], the Speech User Interface Service Quality survey [28], and the Vaccine Confidence Index [29]. The need to standardize evaluation and reporting approaches has also been identified by previous research on health chatbots more generally [10].

### Chatbot User Experience

Several factors were identified in the studies we examined as having a positive influence on users’ perceptions of chatbots. Evidence suggests that providing credible, personalized information in real time through a familiar and accessible platform is key to chatbot success [20]. In addition, making chatbot interactions feel more “natural” by limiting the length of text responses, incorporating images and videos, and eliminating repetition can improve the user experience and engagement [16]. There is also some evidence that anthropomorphic cues, such as the gender of the chatbot persona, can affect how users perceive and engage with chatbots [18]. Conversely, excessively lengthy or repetitious text-based responses, obvious gaps in the knowledge base, and a robotic or inhuman “feel” can all weigh negatively on chatbot user perceptions [16].

While the evidence on factors affecting users’ experience of vaccine chatbots is inevitably limited due to the small number of studies, there are lessons that can potentially be drawn from chatbot evaluations in other health contexts and from chatbot usability research outside the health domain. For example, research suggests that users prefer transparency as to whether they are interacting with an AI system or a human being, as this enables them to calibrate their expectations and their language accordingly [30]. For similar reasons, it is important to be transparent about what tasks the bot can perform and what tasks it cannot, and to offer links to other communication channels in the latter case [30]. While there is some evidence to suggest that users generally prefer audio responses to text-based responses delivered via a screen [31], Alagha and Helbing’s [8] evaluation of voice assistants’ responses to questions about vaccines highlights ethical issues with the “one perfect answer” audio-only approach in a health context, including a lack of transparency around the way answers are generated and the removal of user discretion around the choice of information sources.

### Limitations of the Study

There are several potential limitations to our study. First, we only looked at English-language literature, and therefore it is possible we may have missed relevant studies published in other languages, particularly as data suggest that China currently leads the world in terms of the share of AI journal publications [14]. Second, both the technical aspects of conversational AI and their uses in health care are evolving rapidly. In particular, our literature search predated the public release of ChatGPT in November 2022, which generated significant public interest and likely presages the development of new AI tools for health based on large language models. Indeed, evaluations of ChatGPT in medical contexts have already begun to appear [32]. Thus, while we are confident we have read and addressed all relevant English-language literature available at the time of our literature search, we expect that new interventions and new literature will continue to emerge that may impact our findings and recommendations. Lastly, as highlighted above (see *Results* above) and discussed in more detail below (see *Gaps and Limitations in the Evidence Base* below), there were very few relevant studies at the time of writing the review and each measured different outcomes in different ways, thus limiting our ability to generalize about the impacts of conversational AI for vaccine communication, especially outside of high-income settings.

### Gaps and Limitations in the Evidence Base

Our review identified a number of gaps and limitations in the current literature on conversational AI and vaccine communication. First, the range of vaccines covered and the range of study locations are both very limited, and this could potentially be a source of systemic bias in the evidence base on chatbot effectiveness. We found only one study [20] focusing on vaccines other than COVID-19 and HPV and no studies at all in the global South, which may seem surprising given the incentives to automate aspects of vaccine communication and scheduling in resource-constrained settings where there is a shortage of skilled health workers. However, it is in line with the findings of a recent scoping review on AI in health systems in low- and middle-income countries (LMICs), which identified a number of barriers to adoption, including difficulty integrating AI tools with existing health infrastructure, poor or unstable internet connectivity, and affordability [33]. All of the studies we examined focused on individual chatbots in single study locations. There were no comparative studies that assessed how the effectiveness of chatbots could differ depending on design features and delivery platforms, or between different demographic groups or country locations. In particular, the focus on COVID-19 vaccines as a paradigmatic case study for chatbot evaluation could skew the evidence base for the effectiveness of vaccine chatbots more generally. In theory, chatbots should be most effective at influencing users’ attitudes toward topics where they have little knowledge and few preformed opinions, which would not be the case for many users in relation to COVID-19 vaccines [19].

Second, because chatbot evaluation was largely limited to the short-term, direct effects of chatbot use on users themselves, we know relatively little about the indirect and system-wide effects of a shift toward conversational AI for vaccine communication in the longer term. While respondents in one study indicated a desire to share information they had received through the chatbot [19], none of the studies we examined tried to measure the indirect effects that chatbots might have on nonusers via information sharing. Moreover, the potential longer-term impacts of conversational AI have yet to be analyzed for issues such as information literacy and public trust in health systems. Some experts have expressed concerns that conversational AI, insofar as it is based on the “one perfect answer” ideal or conceals disagreement between information sources, may be less effective at promoting information literacy and critical thinking skills than more traditional information retrieval systems such as search engines [34]. This is an important question because, in the public health sphere, information literacy is widely viewed as an integral component of long-term strategies for building resilience against misinformation and future “infodemics” [35]. Similarly, the potential effects of conversational AI on public trust in health care providers and systems are also unclear but will likely be influenced by public perceptions of chatbots’ usability, reliability, and any “gatekeeping” role that chatbots are perceived to have in relation to health care access [36].

Third, because this is an emerging field of study and many vaccine chatbots are still in the proof-of-concept phase, evaluation has tended to focus on the effectiveness of chatbots rather than their cost-effectiveness. None of the studies we examined provided any information about the costs associated with developing and maintaining chatbots. Consequently, while there is some evidence that chatbots are more effective at improving vaccine attitudes than the same information provided through static text [19,20], the current literature provides no way of assessing whether the marginal benefit of a chatbot outweighs the additional time and resource costs compared to developing a static web page. For the same reason, it is also unclear how far chatbots could be a scalable or sustainable solution to various vaccine communication challenges in the longer term, especially in LMICs [33].

Finally, like the use of AI in health care more generally, vaccine chatbots raise a number of challenges from the ethics perspective that are not adequately addressed in the current literature [36,37]. For instance, we are already seeing the development of chatbots that go beyond simply providing users with accurate and up-to-date vaccine information (on the assumption that this will indirectly influence their vaccine willingness in a positive direction) and instead proactively seek to persuade their users to get a vaccine for themselves or their children. However, for data protection and privacy reasons, it is not generally good practice for chatbots to gather detailed “knowledge” of the individual user’s medical history, religious and cultural beliefs, or the many other personal factors that may be relevant to their vaccine decision-making and would be needed to make a prudent recommendation. In any case, experts have raised doubts about whether conversational AI is, or ever will be, technologically mature enough to replace health professional assessments [36].

### Conclusions and Recommendations

Available evidence, while limited, suggests that conversational AI, properly designed and implemented, can potentially be an effective means of vaccine communication that can complement more traditional channels of health communication, such as consultations with health care providers, especially in situations where health systems are overburdened. While the evidence base on the impact of different chatbot design features remains quite limited, the data in the studies we reviewed does suggest some basic principles that could help maximize the effectiveness of future vaccine chatbots. Specifically, future vaccine chatbots should aim to provide reliable, personalized information in real time through communication platforms that are familiar and accessible to target audiences. So far as possible, chatbot interactions should be designed to emulate the “natural” ebb and flow of human conversation, limit the length of text responses, and incorporate different media such as images and videos. In addition, chatbots focused on childhood immunization need to have the technical capability to tailor the information they provide depending on the child’s age [20].

To conclude, we offer 4 specific recommendations for future research to build the evidence base around conversational AI for vaccine communication and ensure that no unintended harms result from its use.

In the first place, there is a need for further high-quality research on the effectiveness of conversational AI for vaccine communication. There is a particular need for comparative studies that test how chatbot effectiveness may vary depending on design and implementation (eg, anthropomorphic cues, voice or text interfaces), communication context (eg, population-wide or community-specific vaccination campaigns), and across different demographic groups and country locations. Researchers should aim to recruit larger, more representative samples and include control groups. Because studies of this nature are costly, consideration should also be given to enhancing the comparability of studies conducted by research teams working independently of one another through the use of standardized indices of chatbot usability and vaccine attitudes within evaluation questionnaires. Additionally, future interventions should have a stronger theoretical underpinning from behavioral and communication theories such as the Health Belief Model or the Information-Motivation-Behavioral Skills Model.

Second, there is a need to evaluate the longer-term, indirect, and system-wide effects of conversational AI as well as the short-term, direct effects on chatbot users. Since one study found that the relative benefits of chatbot use compared to nonuse declined over time [19], which the authors speculate could be due to nonusers receiving pro-vaccination messaging from other sources during the study period, there would be value in additional longitudinal studies incorporating follow-up surveys of chatbot users and control groups over longer time periods. Where possible, longitudinal surveys should also aim to assess trends in information sharing habits, information literacy, and trust in health care among chatbot users and nonusers over time. Together, these data would help to build the evidence base around the longer-term and indirect effects of conversational AI in this field.

Third, more evidence and transparency around the costs of chatbot development and maintenance are needed, as evaluations currently focus on the communicative benefits of vaccine chatbots without addressing the cost side of the equation. As vaccine communication is still a relatively new application for conversational AI and many chatbots are still in the proof-of-concept stage, it may be premature to expect detailed economic appraisals. However, if future studies could include at least some basic data on the time and resource costs associated with chatbots, this would begin to build an evidence base for the marginal cost-effectiveness of chatbots compared to other forms of vaccine communication, such as web-based FAQs, social media campaigns, webinars, or in-person consultations with health care providers.

Finally, greater consideration needs to be given to how ethics apply in the fields of conversational AI and vaccines. Future research should directly address the question of what may be appropriate or inappropriate tasks for vaccine chatbots to perform based on an analysis of the technical capabilities and limitations of current conversational AI systems. Building this evidence base would enable researchers to make evidence-based recommendations to governments and regulators around appropriate ethical and regulatory frameworks for these technologies in a health context. One interesting avenue of research could be around the technical feasibility and ethical desirability of incorporating relevant ethical frameworks and principles directly into a chatbot’s knowledge base. For the foreseeable future, however, there will be a continuing need for the human designers and researchers of vaccine chatbots to exercise their own ethically informed judgment about prudent and imprudent uses of conversational AI technology.

## Appendix

Multimedia Appendix 1 PRISMA (Preferred Reporting Items for Systematic Reviews and Meta-Analyses) checklist.

## Abbreviations

AI: artificial intelligence
HPV: human papillomavirus
LMIC: low- and middle-income country
NLP: natural language processing

## References

[1] Amiri P, Karahanna E (2022). Chatbot use cases in the COVID-19 public health response. J Am Med Inform Assoc.

[2] Krupp K, Galea J, Madhivanan P, Gerald L (2022). Conversational artificial intelligence: a new approach for increasing influenza vaccination rates in children with asthma?. Vaccine.

[3] Tudor Car Lorainne, Dhinagaran DA, Kyaw BM, Kowatsch T, Joty S, Theng YL, Atun R (2020). Conversational agents in health care: scoping review and conceptual analysis. J Med Internet Res.

[4] Parmar P, Ryu J, Pandya S, Sedoc J, Agarwal S (2022). Health-focused conversational agents in person-centered care: a review of apps. NPJ Digit Med.

[5] Weizenbaum J (1966). ELIZA-a computer program for the study of natural language communication between man and machine. Commun. ACM.

[6] Adamopoulou E, Moussiades L (2020). Chatbots: history, technology, and applications. Mach Learn Appl.

[7] World Health Organization. Infodemic.

[8] Alagha EC, Helbing RR (2019). Evaluating the quality of voice assistants' responses to consumer health questions about vaccines: an exploratory comparison of Alexa, Google Assistant and Siri. BMJ Health Care Inform.

[9] Boyd M, Wilson N (2018). Just ask Siri? A pilot study comparing smartphone digital assistants and laptop Google searches for smoking cessation advice. PLoS One.

[10] Laranjo L, Dunn AG, Tong HL, Kocaballi AB, Chen J, Bashir R, Surian D, Gallego B, Magrabi F, Lau AYS, Coiera E (2018). Conversational agents in healthcare: a systematic review. J Am Med Inform Assoc.

[11] Montenegro JLZ, da Costa CA, da Rosa Righi R (2019). Survey of conversational agents in health. Expert Syst Appl.

[12] Wilson N, MacDonald EJ, Mansoor OD, Morgan J (2017). In bed with Siri and Google Assistant: a comparison of sexual health advice. BMJ.

[13] Bérubé C, Kovacs ZF, Fleisch E, Kowatsch T (2021). Reliability of commercial voice assistants' responses to health-related questions in noncommunicable disease management: factorial experiment assessing response rate and source of information. J Med Internet Res.

[14] (2023). The AI index report. Measuring trends in artificial Intelligence. Stanford University.

[15] Page MJ, McKenzie JE, Bossuyt PM, Boutron I, Hoffmann TC, Mulrow CD, Shamseer L, Tetzlaff JM, Akl EA, Brennan SE, Chou R, Glanville J, Grimshaw JM, Hróbjartsson A, Lalu MM, Li T, Loder EW, Mayo-Wilson E, McDonald S, McGuinness LA, Stewart LA, Thomas J, Tricco AC, Welch VA, Whiting P, Moher D (2021). The PRISMA 2020 statement: an updated guideline for reporting systematic reviews. BMJ.

[16] Amith M, Zhu A, Cunningham R, Lin R, Savas L, Shay L, Chen Y, Gong Y, Boom J, Roberts K, Tao C (2019). Early usability assessment of a conversational agent for HPV vaccination. Stud Health Technol Inform.

[17] Amith M, Lin R, Cunningham R, Wu QL, Savas LS, Gong Y, Boom JA, Tang L, Tao C (2020). Examining potential usability and health beliefs among young adults using a conversational agent for HPV vaccine counseling. AMIA Jt Summits Transl Sci Proc.

[18] Tsai WHS, Lun D, Carcioppolo N, Chuan CH (2021). Human versus chatbot: understanding the role of emotion in health marketing communication for vaccines. Psychol Mark.

[19] Altay S, Hacquin AS, Chevallier C, Mercier H (2023). Information delivered by a chatbot has a positive impact on COVID-19 vaccines attitudes and intentions. J Exp Psychol Appl.

[20] Hong YJ, Piao M, Kim J, Lee JH (2021). Development and evaluation of a child vaccination chatbot real-time consultation messenger service during the COVID-19 pandemic. Appl Sci.

[21] Kobayashi T, Nishina Y, Tomoi H, Harada K, Tanaka K, Matsumoto E, Horimukai K, Ishihara J, Sasaki S, Inaba K, Seguchi K, Takahashi H, Salinas JL, Yamada Y (2022). Corowa-kun: a messenger app chatbot delivers COVID-19 vaccine information, Japan 2021. Vaccine.

[22] Chalaguine L, Hunter A, Vejnarová J, Wilson N (2021). Addressing popular concerns regarding COVID-19 vaccination with natural language argumentation dialogues. Symbolic and Quantitative Approaches to Reasoning with Uncertainty.

[23] Attribution 4.0 International (CC BY 4.0). Creative Commons.

[24] Attwell K, Freeman M (2015). I Immunise: an evaluation of a values-based campaign to change attitudes and beliefs. Vaccine.

[25] Pluviano S, Watt C, Della Sala S (2017). Misinformation lingers in memory: failure of three pro-vaccination strategies. PLoS One.

[26] Zollo F (2019). Dealing with digital misinformation: a polarised context of narratives and tribes. EFSA J.

[27] Holmes S, Moorhead A, Bond R, Zheng H, Coates V, Mctear M (2019). Usability testing of a healthcare chatbot: can we use conventional methods to assess conversational user interfaces?. https://dl.acm.org/doi/proceedings/10.1145/3335082.

[28] Polkosky MD, Konijn EA, Utz S, Tanis M, Barnes SB (2008). The challenge of technology for interpersonal communication theory and research. Machines as Mediators, 1st Edition.

[29] Larson HJ, Schulz WS, Tucker JD, Smith DMD (2015). Measuring vaccine confidence: introducing a global vaccine confidence index. PLoS Curr.

[30] Budiu R (2018). The user experience of chatbots. Nielsen Norman Group.

[31] Budiu R, Laubheimer P Intelligent assistants have poor usability: a user study of Alexa, Google Assistant, and Siri. Nielsen Norman Group.

[32] Ayers JW, Poliak A, Dredze M, Leas EC, Zhu Z, Kelley JB, Faix DJ, Goodman AM, Longhurst CA, Hogarth M, Smith DM (2023). Comparing physician and artificial intelligence chatbot responses to patient questions posted to a public social media forum. JAMA Intern Med.

[33] Ciecierski-Holmes T, Singh R, Axt M, Brenner S, Barteit S (2022). Artificial intelligence for strengthening healthcare systems in low- and middle-income countries: a systematic scoping review. NPJ Digit Med.

[34] Shah C, Bender EM (2022). Situating Search. https://dl.acm.org/doi/proceedings/10.1145/3498366.

[35] Naeem SB, Boulos MNK (2021). COVID-19 misinformation online and health literacy: a brief overview. Int J Environ Res Public Health.

[36] Parviainen J, Rantala J (2022). Chatbot breakthrough in the 2020s? An ethical reflection on the trend of automated consultations in health care. Med Health Care Philos.

[37] Xu L, Sanders L, Li K, Chow JCL (2021). Chatbot for health care and oncology applications using artificial intelligence and machine learning: systematic review. JMIR Cancer.

